# On a Family of Multivariate Modified Humbert Polynomials

**DOI:** 10.1155/2013/187452

**Published:** 2013-07-14

**Authors:** Rabia Aktaş, Esra Erkuş-Duman

**Affiliations:** ^1^Department of Mathematics, Faculty of Science, Ankara University, Tandoğan, 06100 Ankara, Turkey; ^2^Department of Mathematics, Faculty of Science, Gazi University, Teknikokullar, 06500 Ankara, Turkey

## Abstract

This paper attempts to present a multivariable extension of generalized Humbert polynomials. The results obtained here include various families of multilinear and multilateral generating functions, miscellaneous properties, and also some special cases for these multivariable polynomials.

## 1. Introduction

The generalized Humbert polynomials are generated by [1]

(1) $$\begin{array}{c} {\left(C-mxt+yt^{m}\right)}^{p}=\sum_{n=0}^{\infty }P_{n}\left(m,x,y,p,C\right)t^{n}, \end{array}$$

where *m* is a positive integer and other parameters are unrestricted in general (see also [2, pages 77, 86] and [3, 4]). This definition includes many well-known special polynomials such as Humbert, Louville, Gegenbauer, Legendre, Tchebycheff, Pincherle, and Kinney polynomials.

In this paper, we consider the following multivariable extension of the generalized Humbert polynomials which are completely different from the polynomials introduced in [5]. This class of polynomials is generated by

(2) (1−∑i=1r(mixiti−yitimi))−α  =∑n1,…,nr=0∞Θn1,…,nr(α)(x,y,m)t1n1⋯trnr,     (|∑i=1r(mixiti−yitimi)|<1),

where **x** = (*x*_1_,…, *x*_*r*_),  **y** = (*y*_1_,…, *y*_*r*_), **m** = (*m*_1_,…, *m*_*r*_) and *m*_*i*_  (*i* = 1,2,…, *r*) is a positive integer. It follows from (2) that

(3) Θn1,…,nr(α)(x,y,m) =∑k1=0[n1/m1]⋯∑kr=0[nr/mr](α)n1+⋯+nr−(m1−1)k1−⋯−(mr−1)krk1!⋯kr!(n1−m1k1)!⋯(nr−mrkr)!  ×(−1)k1+⋯+kr(m1x1)n1−m1k1⋯(mrxr)nr−mrkry1k1⋯yrkr,

where (*λ*)_*k*_ : = *λ*(*λ* + 1) ⋯ (*λ* + *k* − 1), (*λ*)_0_ : = 1 is the Pochhammer symbol.

The aim of this paper is to derive various families of multilinear and multilateral generating functions and to give several recurrence relations and expansions in the series of orthogonal polynomials for the family of multivariable polynomials given explicitly by (3). We present some special cases of our results and also obtain some other properties for these special cases.

## 2. Bilinear and Bilateral Generating Functions

In this section, with the help of the similar method as considered in [5–9], we derive several families of bilinear and bilateral generating functions for the family of multivariable polynomials generated by (2) and given explicitly by (3).

We begin by stating the following theorem.

**Theorem 1**

Corresponding to an identically nonvanishing function *Ω*_*μ*_(**z**) of *s* complex variables *z*_1_,…, *z*_*s*_  (*s* ∈ *ℕ*) and of complex order *μ*, let

(4) $$\begin{array}{c} \Lambda_{\mu ,\nu }\left(z;w\right):=\sum_{k=0}^{\infty }a_{k}\Omega_{\mu +\nu k}\left(z\right)w^{k}, \end{array}$$

where (*a*_*k*_ ≠ 0,   *μ*, *ν* ∈ *ℂ*); **z** = (*z*_1_,…, *z*_*s*_). Then, for *p* ∈ *ℕ*; **x** = (*x*_1_,…, *x*_*r*_); **y** = (*y*_1_,…, *y*_*r*_); **m** = (*m*_1_,…, *m*_*r*_), *m*_*i*_ ∈ *ℕ* (*i* = 1,2,…, *r*), one has

(5) ∑n1,…,nr=0∞∑k=0[n1/p]  akΘn1−pk,n2,…,nr(α)(x,y,m)       ×t2n2⋯trnrΩμ+νk(z)ηkt1n1−pk  =(1−∑i=1r(mixiti−yitimi))−αΛμ,ν(z;η)

provided that each member of (5) exists.

**Proof**

For convenience, let *S* denote the first member of the assertion (5) of Theorem 1. Replacing *n*_1_ by *n*_1_ + *pk*, we may write that

(6) S=∑n1,…,nr=0∞ ‍∑k=0∞ak  Θn1,n2,…,nr(α)(x,y,m)Ωμ+νk(z)ηkt1n1⋯trnr=∑n1,…,nr=0∞Θn1,n2,…,nr(α)(x,y,m)t1n1⋯trnr∑k=0∞akΩμ+νk(z)ηk=(1−∑i=1r(mixiti−yitimi))−αΛμ,ν(z;η),

which completes the proof.

By using a similar idea, we also get the next result immediately.

**Theorem 2**

For a nonvanishing function *φ*_*λ*_1_,…,*λ*_*r*__(**z**) of *s* complex variables *z*_1_,…, *z*_*s*_  (*s* ∈ *ℕ*) and for *p*_*i*_ ∈ *ℕ*, *λ*_*i*_, *η*_*i*_ ∈ *ℂ*, **z** = (*z*_1_,…, *z*_*s*_), **w** = (*w*_1_,…, *w*_*r*_), *λ* : = (*λ*_1_,…, *λ*_*r*_),  *η* : = (*η*_1_,…, *η*_*r*_) let

(7) Ωλ,η,α,β,mn,p(x,y;z;w) :=∑k1=0[n1/p1]⋯∑kr=0[nr/pr]ak1,…,krΘn1−p1k1,…,nr−prkr(α+β)(x,y,m)          ×φλ1+η1k1,…,λr+ηrkr(z)w1k1⋯wrkr,

where *a*_*k*_1_,…,*k*_*r*__ ≠ 0; *n*_*i*_, (*i* = 1,2,…, *r*) ∈ *ℕ*_0_; *ℕ*_0_ : = *ℕ* ∪ {0}. Then, one has

(8) ∑l1=0n1⋯∑lr=0nr∑k1=0[l1/p1]⋯∑kr=0[lr/pr]ak1,…,krΘn1−l1,…,nr−lr(α)(x,y,m)×Θl1−p1k1,…,lr−prkr(β)(x,y,m)×φλ1+η1k1,…,λr+ηrkr(z)×w1k1⋯wrkr =Ωλ,η,α,β,mn,p(x,y;z;w)

provided that each member of (8) exists.

## 3. Special Cases

As an application of the above theorems, when the multivariable function *Ω*_*μ*+*νk*_(**z**),  **z** = (*z*_1_,…, *z*_*s*_),  *k* ∈ *ℕ*_0_, *s* ∈ *ℕ*, is expressed in terms of simpler functions of one and more variables, then we can give further applications of the above theorems. We first set

(9) $$\begin{array}{c} s=r,\;\;\Omega_{\mu +\nu k}\left(z\right)={\text{g}}_{\mu +\nu k}^{\left(\beta_{1},\ldots ,\beta_{r}\right)}\left(z\right) \end{array}$$

in Theorem 1, where the Chan-Chyan-Srivastava polynomials g_*μ*+*νk*_^(*β*_1_,…,*β*_*r*_)^(**z**) [10] are generated by

(10) ∏i=1r(1−xit)−αi=∑n=0∞gn(α1,…,αr)(x1,…,xr)tn(αi∈ℂ(i=1,2,…,r);|t|<min⁡{|x1|−1,…,|xr|−1}).

We are thus led to the following result which provides a class of bilateral generating functions for the Chan-Chyan-Srivastava polynomials and the family of multivariable polynomials given explicitly by (3).

**Corollary 3**

If Λ_*μ*,*ν*_(**z**; *w*): = ∑_*k*=0_^*∞*^ *a*_*k*_ g _*μ*+*νk*_^(*β*_1_,…,*β*_*r*_)^(**z**)*w* ^*k*^, *a*_*k*_ ≠ 0, *μ*, *ν* ∈ *ℂ*, **z** = (*z*_1_,…, *z*_*r*_) then, one has

(11) ∑n1,…,nr=0∞∑k=0[n1/p]akΘn1−pk,n2,…,nr(α)(x,y,m)×t2n2⋯trnr 
g
 μ+νk(β1,…,βr)(z)ηkt1n1−pk=(1−∑i=1r(mixiti−yitimi))−αΛμ,ν(z;η)

provided that each member of (11) exists.

**Remark 4**

Using the generating relation (10) for the Chan-Chyan-Srivastava polynomials and getting *a*_*k*_ = 1,  *μ* = 0, *ν* = 1, we find that

(12) ∑n1,…,nr=0∞ ∑k=0[n1/p]Θn1−pk,n2,…,nr(α)(x,y,m)t2n2⋯trnrgk(β1,…,βr)(z)×ηkt1n1−pk  =(1−∑i=1r(mixiti−yitimi))−α∏i=1r(1−ziη)−βi,|η|<min⁡{|z1|−1,…,|zr|−1},|∑i=1r(mixiti−yitimi)|<1.

On the other hand, choosing *s* = 2*r*  and

(13) $$\begin{array}{c} \varphi_{\lambda_{1}+\eta_{1}k_{1},\ldots ,\lambda_{r}+\eta_{r}k_{r}}\left(z\right)=\Theta_{\lambda_{1}+\eta_{1}k_{1},\ldots ,\lambda_{r}+\eta_{r}k_{r}}^{\left(\gamma \right)}\left(u,v,m\right), \end{array}$$

*λ*_*i*_, *η*_*i*_  (*i* = 1,2,…, *r*) ∈ *ℕ*_0_,  **u** = (*u*_1_,…, *u*_*r*_), **v** = (*v*_1_,…, *v*_*r*_) in Theorem 2, we obtain the following class of bilinear generating functions for the family of multivariable polynomials given explicitly by (3).

**Corollary 5**

If

(14) Λλ,η,α,β,γ,mn,p(x,y;u,v;w) :=∑k1=0[n1/p1]⋯∑kr=0[nr/pr]ak1,…,krΘn1−p1k1,…,nr−prkr(α+β)(x,y,m)  ×Θλ1+η1k1,…,λr+ηrkr(γ)(u,v,m)w1k1⋯wrkr,

where *a*_*k*_1_,…,*k*_*r*__ ≠ 0; *p*_*i*_ ∈ *ℕ*; *n*_*i*_, *λ*_*i*_, *η*_*i*_ ∈ *ℕ*_0_, *i* = 1,2,…, *r*, then, we get

(15) ∑l1=0n1⋯∑lr=0nr ‍ ∑k1=0[l1/p1]⋯∑kr=0[lr/pr]ak1,…,krΘn1−l1,…,nr−lr(α)(x,y,m)×Θl1−p1k1,…,lr−prkr(β)(x,y,m)×Θλ1+η1k1,…,λr+ηrkr(γ)(u,v,m)×w1k1⋯wrkr  =Λλ,η,α,β,γ,mn,p(x,y;u,v;w)

provided that each member of (15) exists.

Furthermore, for every suitable choice of the coefficients *a*_*k*_  (*k* ∈ *ℕ*_0_), if the multivariable functions *Ω*_*μ*+*νk*_(**y**) and *φ*_*λ*_1_+*η*_1_*k*_1_,…,*λ*_*r*_+*η*_*r*_*k*_*r*__(**y**),  **y** = (*y*_1_,…, *y*_*s*_), (*s* ∈ *ℕ*), are expressed as an appropriate product of several simpler functions, the assertions of Theorems 1 and 2 can be applied in order to derive various families of multilinear and multilateral generating functions for the family of multivariable polynomials given explicitly by (3).

## 4. Some Miscellaneous Properties

In this section, we now discuss some further properties of the family of multivariable polynomials given by (3). We start with the following theorems.

**Theorem 6**

Let Ψ_*n*_1_,…,*n*_*r*__(**x**, **y**, **m**) be a family of functions generated by

(16) G(m1x1t1−y1t1m1,…,mrxrtr−yrtrmr)  =∑n1,…,nr=0∞Ψn1,…,nr(x,y,m)t1n1⋯trnr.

The following relations hold:

(17) niΨn1,…,nr(x,y,m)=xi∂∂xiΨn1,…,nr(x,y,m) −yi∂∂xiΨn1,…,ni−1,ni−mi+1,ni+1,…,nr(x,y,m)

for *n*_*i*_ ≥ *m*_*i*_ − 1,  *i* = 1,2,…, *r*; *n*_*j*_ ≥ 0,  *j* ≠ *i* and

(18) $$\begin{array}{c} n_{i}\Psi_{n_{1},\ldots ,n_{r}}\left(x,y,m\right)=x_{i}\frac{\partial }{\partial x_{i}}\Psi_{n_{1},\ldots ,n_{r}}\left(x,y,m\right) \end{array}$$

for *n*_*i*_ = 0,1,…, *m*_*i*_ − 2, *i* = 1,2,…, *r*; *n*_*j*_ ≥ 0, *j* ≠ *i*. Also, one finds that

(19) mixi∂∂yiΨn1,…,nr(x,y,m)−miyi∂∂yi×Ψn1,…,ni−1,ni−mi+1,ni+1,…,nr(x,y,m)=−(ni−mi+1)Ψn1,…,ni−1,ni−mi+1,ni+1,…,nr(x,y,m)

for *n*_*i*_ ≥ *m*_*i*_ − 1, *i* = 1,2,…, *r*; *n*_*j*_ ≥ 0, *j* ≠ *i* and

(20) $$\begin{array}{c} \frac{\partial }{\partial y_{i}}\Psi_{n_{1},\ldots ,n_{r}}\left(x,y,m\right)=0 \end{array}$$

for *n*_*i*_ = 0,1,…, *m*_*i*_ − 2,  *i* = 1,2,…, *r*; *n*_*j*_ ≥ 0, *j* ≠ *i*.

**Proof**

Fix *i* = 1,2,…, *r*. Then, by differentiating (16) with respect to *x*_*i*_ and *t*_*i*_, after making necessary calculations we obtain that

(21) ∑n1,…,nr=0∞niΨn1,…,nr(x,y,m)t1n1⋯trnr =(xi−yitimi−1)∑n1,…,nr=0∞∂∂xiΨn1,…,nr(x,y,m)t1n1⋯trnr =xi∑n1,…,nr=0∞∂∂xiΨn1,…,nr(x,y,m)t1n1⋯trnr  −yi∑n1,…,ni−1,ni+1,…,nr=0(ni≥mi−1)∞∂∂xiΨn1,…,ni−1,ni−mi+1,ni+1,…,nr(x,y,m)×t1n1⋯trnr.

Comparing the coefficients of *t*_1_^*n*_1_^ ⋯ *t*_*r*_^*n*_*r*_^, we obtain (17) and (18) for the fixed *i*. Similarly, if we differentiate (16) with respect to *y*_*i*_ and *t*_*i*_, we can find the relation (19).

**Theorem 7**

If Ψ_*n*_1_,…,*n*_*r*__(**x**, **y**, **m**) is a family of functions generated by (16), then it satisfies the relations

(22) −mi∂∂yiΨn1,…,ni−1,ni−1,ni+1,…,nr(x,y,m)  =∂∂xiΨn1,…,ni−1,ni−mi,ni+1,…,nr(x,y,m)

for *n*_*i*_ ≥ *m*_*i*_, *i* = 1,2,…, *r*; *n*_*j*_ ≥ 0, *j* ≠ *i* and

(23) $$\begin{array}{c} \frac{\partial }{\partial y_{i}}\Psi_{n_{1},\ldots ,n_{i-1},n_{i}-1,n_{i+1},\ldots ,n_{r}}\left(x,y,m\right)=0 \end{array}$$

for *n*_*i*_ = 1,2,…, *m*_*i*_ − 1, *i* = 1,2,…, *r*; *n*_*j*_ ≥ 0, *j* ≠ *i*.

**Proof**

By comparing the derivatives of (16) with respect to *x*_*i*_ and *y*_*i*_, we have

(24) −miti∑n1,…,nr=0∞∂∂yiΨn1,…,nr(x,y,m)t1n1⋯trnr  =timi∑n1,…,nr=0∞∂∂xiΨn1,…,nr(x,y,m)t1n1⋯trnr,

which implies that

(25) −mi∂∂yiΨn1,…,ni−1,ni−1,ni+1,…,nr(x,y,m)  =∂∂xiΨn1,…,ni−1,ni−mi,ni+1,…,nr(x,y,m)

for *n*_*i*_ ≥ *m*_*i*_, *i* = 1,2,…, *r*; *n*_*j*_ ≥ 0, *j* ≠ *i* and

(26) $$\begin{array}{c} \frac{\partial }{\partial y_{i}}\Psi_{n_{1},\ldots ,n_{i-1},n_{i}-1,n_{i+1},\ldots ,n_{r}}\left(x,y,m\right)=0 \end{array}$$

for *n*_*i*_ = 1,2,…, *m*_*i*_ − 1,  *i* = 1,2,…, *r*; *n*_*j*_ ≥ 0, *j* ≠ *i*. Thus, the proof is completed.

As a result of these theorems, if we choose

(27) G(m1x1t1−y1t1m1,…,mrxrtr−yrtrmr)  =(1−∑i=1r(mixiti−yitimi))−α,

then we can give some recurrence relations for the family of multivariable polynomials given explicitly by (3). In the view of Theorem 6, we get

**Corollary 8**

For the family of multivariable polynomials generated by (2), the following relations hold

(28) niΘn1,…,nr(α)(x,y,m)=xi∂∂xiΘn1,…,nr(α)(x,y,m)−yi∂∂xiΘn1,…,ni−1,ni−mi+1,ni+1,…,nr(α)(x,y,m),

(29) mixi∂∂yiΘn1,…,nr(α)(x,y,m)−miyi∂∂yi×Θn1,…,ni−1,ni−mi+1,ni+1,…,nr(α)(x,y,m)=−(ni−mi+1)Θn1,…,ni−1,ni−mi+1,ni+1,…,nr(α)(x,y,m)

for *n*_*i*_ ≥ *m*_*i*_ − 1,  *i* = 1,2,…, *r*; *n*_*j*_ ≥ 0, *j* ≠ *i*. Also, for *n*_*i*_ = 0,1,…, *m*_*i*_ − 2, *i* = 1,2,…, *r*; *n*_*j*_ ≥ 0, *j* ≠ *i*, we have

(30) $$\begin{array}{c} n_{i}\Theta_{n_{1},\ldots ,n_{r}}^{\left(\alpha \right)}\left(x,y,m\right)=x_{i}\frac{\partial }{\partial x_{i}}\Theta_{n_{1},\ldots ,n_{r}}^{\left(\alpha \right)}\left(x,y,m\right), \end{array}$$

(31) $$\begin{array}{c} \frac{\partial }{\partial y_{i}}\Theta_{n_{1},\ldots ,n_{r}}^{\left(\alpha \right)}\left(x,y,m\right)=0. \end{array}$$

**Corollary 9**

By summing the relations given by (28) and (29), respectively, for *i* = 1,2,…, *r*, we get

(32) ∑i=1rniΘn1,…,nr(α)(x,y,m)  =∑i=1rxi∂∂xiΘn1,…,nr(α)(x,y,m)   −∑i=1ryi∂∂xiΘn1,…,ni−1,ni−mi+1,ni+1,…,nr(α)(x,y,m),∑i=1rmixi∂∂yiΘn1,…,nr(α)(x,y,m)   −∑i=1rmiyi∂∂yiΘn1,…,ni−1,ni−mi+1,ni+1,…,nr(α)(x,y,m)   =−∑i=1r(ni−mi+1)       ×Θn1,…,ni−1,ni−mi+1,ni+1,…,nr(α)(x,y,m)

for *n*_*i*_ ≥ *m*_*i*_ − 1, *i* = 1,2,…, *r*; *n*_*j*_ ≥ 0, *j* ≠ *i*.

Similarly, as a consequence of Theorem 7, we can give the next result at once.

**Corollary 10**

Other recurrence relations for the family of multivariable polynomials Θ_*n*_1_,…,*n*_*r*__^(*α*)^(**x**, **y**, **m**) are

(33) −mi∂∂yiΘn1,…,ni−1,ni−1,ni+1,…,nr(α)(x,y,m)  =∂∂xiΘn1,…,ni−1,ni−mi,ni+1,…,nr(α)(x,y,m)

for *n*_*i*_ ≥ *m*_*i*_, *i* = 1,2,…, *r*; *n*_*j*_ ≥ 0, *j* ≠ *i* and

(34) $$\begin{array}{c} \frac{\partial }{\partial y_{i}}\Theta_{n_{1},\ldots ,n_{i-1},n_{i}-1,n_{i+1},\ldots ,n_{r}}^{\left(\alpha \right)}\left(x,y,m\right)=0 \end{array}$$

for *n*_*i*_ = 1,2,…, *m*_*i*_ − 1, *i* = 1,2,…, *r*; *n*_*j*_ ≥ 0, *j* ≠ *i*.

**Theorem 11**

The generating relation (2) yields the following addition formula for the family of multivariable polynomials given by (3)

(35) Θn1,…,nr(α+β)(x,y,m)=∑k1=0n1⋯∑kr=0nrΘn1−k1,…,nr−kr(α)(x,y,m)×Θk1,…,kr(β)(x,y,m).

**Proof**

From (2), we have

(36) ∑n1,…,nr=0∞Θn1,…,nr(α+β)(x,y,m)t1n1⋯trnr  =(1−∑i=1r(mixiti−yitimi))−(α+β)  =(1−∑i=1r(mixiti−yitimi))−α   ×(1−∑i=1r(mixiti−yitimi))−β  =∑n1,…,nr=0∞Θn1,…,nr(α)(x,y,m)t1n1⋯trnr   ×∑k1,…,kr=0∞Θk1,…,kr(β)(x,y,m)t1k1⋯trkr.

Replacing *n*_*i*_ by *n*_*i*_ − *k*_*i*_, *i* = 1,2,…, *r*,   the right-hand side of the last equality is

(37) ∑n1,…,nr=0∞ ‍∑k1=0n1⋯∑kr=0nrΘn1−k1,…,nr−kr(α)(x,y,m)×Θk1,…,kr(β)(x,y,m)t1n1⋯trnr.

Comparing the coefficients of *t*_1_^*n*_1_^ ⋯ *t*_*r*_^*n*_*r*_^ completes the proof.

We now give expansions of the family of multivariable polynomials Θ_*n*_1_,…,*n*_*r*__^(*α*)^(**x**, **y**, **m**) given explicitly by (3) in series of Legendre, Gegenbauer, Hermite, and Laguerre polynomials.

**Theorem 12**

Expansions of Θ_*n*_1_,…,*n*_*r*__^(*α*)^(**x**, **y**, **m**) in series of Legendre, Gegenbauer, Hermite, and Laguerre polynomials are as follows

(38) Θn1,…,nr(α)(x,y,m) =∏i=1r{∑ki=0[ni/mi] ∑si=0[(ni−miki)/2](2ni−2miki−4si+1)ki!si!(3/2)ni−miki−si     ×(−1)kiyikiPni−miki−2si(mixi2)}    ×(α)n1+⋯+nr−(m1−1)k1−⋯−(mr−1)kr,Θn1,…,nr(α)(x,y,m) =∏i=1r{∑ki=0[ni/mi] ∑si=0[(ni−miki)/2](νi+ni−miki−2si)ki!si!(νi)ni−miki−si+1     ×(−yi)kiCni−miki−2siνi(mixi2)}   ×(α)n1+⋯+nr−(m1−1)k1−⋯−(mr−1)kr,Θn1,…,nr(α)(x,y,m) =∏i=1r{∑ki=0[ni/mi] ∑si=0[(ni−miki)/2](−yi)kiHni−miki−2si(mixi/2)ki!si!(ni−miki−2si)!}   ×(α)n1+⋯+nr−(m1−1)k1−⋯−(mr−1)kr,Θn1,…,nr(α)(x,y,m) =∏i=1r{∑ki=0[ni/mi] ‍∑si=0ni−miki(βi+1)ni−miki2ni−miki(−1)siki!(ni−miki−si)!(βi+1)si     ×(−yi)kiLsi(βi)(mixi2)}    ×(α)n1+⋯+nr−(m1−1)k1−⋯−(mr−1)kr.

**Proof**

By (2), we get

(39) ∑n1,…,nr=0∞Θn1,…,nr(α)(x,y,m)t1n1⋯trnr =(1−∑i=1r(mixiti−yitimi))−α =∑n1,…,nr=0∞ ‍∑k1,…,kr=0∞(α)n1+⋯+nr+k1+⋯+krk1!⋯kr!n1!⋯nr!(−1)k1+⋯+kr×(m1x1)n1⋯(mrxr)nry1k1⋯yrkr×t1n1+m1k1⋯trnr+mrkr.

If we use the result in [11, page 181]

(40) $$\begin{array}{c} \frac{{\left(mx\right)}^{n}}{n!}=\sum_{s=0}^{\left[n/2\right]}\frac{\left(2n-4s+1\right)P_{n-2s}\left(mx/2\right)}{s!{\left(3/2\right)}_{n-s}}, \end{array}$$

we can write that

(41) ∑n1,…,nr=0∞Θn1,…,nr(α)(x,y,m)t1n1⋯trnr =∏i=1r{∑ni=0∞ ‍∑ki=0∞ ‍∑si=0[ni/2](2ni−4si+1)si!ki!(3/2)ni−siPni−2si(mixi2)×(−yi)kitini+miki}(α)n1+⋯+nr+k1+⋯+kr.

Getting *n*_*i*_ − *m*_*i*_ *k*_*i*_ instead of *n*_*i*_ in the last equality, we have

(42) ∑n1,…,nr=0∞Θn1,…,nr(α)(x,y,m)t1n1⋯trnr =∑n1,…,nr=0∞ ‍∏i=1r{∑ki=0[ni/mi] ∑si=0[(ni−miki)/2](2ni−2miki−4si+1)si!ki!(3/2)ni−miki−si          ×Pni−miki−2si(mixi2)(−yi)kitini}       ×(α)n1+⋯+nr−(m1−1)k1−⋯−(mr−1)kr =∑n1,…,nr=0∞{∏i=1r∑ki=0[ni/mi] ∑si=0[(ni−miki)/2](2ni−2miki−4si+1)si!ki!(3/2)ni−miki−si        ×Pni−miki−2si(mixi2)(−yi)ki}     ×(α)n1+⋯+nr−(m1−1)k1−⋯−(mr−1)krt1n1⋯trnr.

Comparing the coefficients of *t*_1_^*n*_1_^ ⋯ *t*_*r*_^*n*_*r*_^ gives the desired relation.

In a similar manner, in (39), using the following results, respectively, [11, page 283 (36), page 194 (4), page 207 (2)]

(43) $$\begin{array}{c} \frac{{\left(mx\right)}^{n}}{n!}=\sum_{k=0}^{\left[n/2\right]}\frac{\left(\nu +n-2k\right)C_{n-2k}^{\nu }\left(mx/2\right)}{k!{\left(\nu \right)}_{n+1-k}},\\ \frac{{\left(mx\right)}^{n}}{n!}=\sum_{k=0}^{\left[n/2\right]}\frac{H_{n-2k}\left(mx/2\right)}{k!\left(n-2k\right)!},\\ \frac{{\left(mx\right)}^{n}}{n!}=2^{n}\sum_{k=0}^{n}\frac{{\left(-1\right)}^{k}{\left(\alpha +1\right)}_{n}L_{k}^{\left(\alpha \right)}\left(mx/2\right)}{\left(n-k\right)!{\left(\alpha +1\right)}_{k}}, \end{array}$$

one can easily obtain the other expansions of Θ_*n*_1_,…,*n*_*r*__^(*α*)^(**x**, **y**, **m**) in series of Gegenbauer, Hermite, and Laguerre polynomials.

## 5. The Special Cases of Θ_*n*_^(*α*)^ (x, y, m) and Some Properties

In this section, we discuss some special cases of the family of multivariable polynomials Θ_*n*_^(*α*)^(**x**, **y**, **m**) and give their several properties.

### 5.1. The Case of *m*_*i*_ = 2, *y*_*i*_ = 1, *i* = 1,2,…, *r* in (2)

This case gives a multivariable analogue of Gegenbauer polynomials

(44) (1−2x1t1+t12−⋯−2xrtr+tr2)−α  =∑n1,…,nr=0∞Cn1,…,nr(α)(x1,…,xr)t1n1⋯trnr,

which reduces to two variables Gegenbauer polynomials given by [12] for *r* = 2. Equation (44) yields the following formula:

(45) Cn1,…,nr(α)(x1,…,xr) =∑k1=0[n1/2]⋯∑kr=0[nr/2](((α)n1+⋯+nr−k1−⋯−kr)×(k1!⋯kr!(n1−2k1)!⋯(nr−2kr)!)−1)×(−1)k1+⋯+kr(2x1)n1−2k1⋯(2xr)nr−2kr.

It follows that *C*_*n*_1_,…,*n*_*r*__^(*α*)^(*x*_1_,…, *x*_*r*_) is a polynomial of degree *n*_*i*_ with respect to the fixed variable *x*_*i*_  (*i* = 1,2,…, *r*).   Thus, *C*_*n*_1_,…,*n*_*r*__^(*α*)^(*x*_1_,…, *x*_*r*_) is a polynomial of total degree (*n*_1_ + ⋯+*n*_*r*_) with respect to the variables *x*_1_,…, *x*_*r*_. Equation (45) also yields

(46) Cn1,…,nr(α)(x1,…,xr)  =2n1+⋯+nr(α)n1+⋯+nrn1!⋯nr!x1n1⋯xrnr+Π(x1,…,xr),

where Π(*x*_1_,…, *x*_*r*_) is a polynomial of degree (*n*_1_ + ⋯+*n*_*r*_ − 2) with respect to the variables *x*_1_,…, *x*_*r*_. In (44), by getting *x*_1_ → −*x*_1_ and *t*_1_ → −*t*_1_, we have

(47) $$\begin{array}{c} C_{n_{1},\ldots ,n_{r}}^{\left(\alpha \right)}\left(-x_{1},x_{2},\ldots ,x_{r}\right)={\left(-1\right)}^{n_{1}}C_{n_{1},\ldots ,n_{r}}^{\left(\alpha \right)}\left(x_{1},\ldots ,x_{r}\right). \end{array}$$

Similarly, for *i* = 1,2,…, *r*,   we get

(48) Cn1,…,nr(α)(x1,…,xi−1,−xi,xi+1,…,xr)  =(−1)niCn1,…,nr(α)(x1,…,xr).

Taking *x*_*i*_ → −*x*_*i*_ and  *t*_*i*_ → −*t*_*i*_, *i* = 1,2,…, *r*, in (44), we obtain

(49) $$\begin{array}{c} C_{n_{1},\ldots ,n_{r}}^{\left(\alpha \right)}\left(-x_{1},\ldots ,-x_{r}\right)={\left(-1\right)}^{n_{1}+\cdots +n_{r}}C_{n_{1},\ldots ,n_{r}}^{\left(\alpha \right)}\left(x_{1},\ldots ,x_{r}\right). \end{array}$$

**Theorem 13**

For the polynomials *C*_*n*_1_,…,*n*_*r*__^(*α*)^(*x*_1_,…, *x*_*r*_), one has

(50) $$\begin{array}{c} C_{2n_{1},\ldots ,2n_{r}}^{\left(\alpha \right)}\left(0,0,\ldots ,0\right)=\frac{{\left(-1\right)}^{n_{1}+\cdots +n_{r}}{\left(\alpha \right)}_{n_{1}+\cdots +n_{r}}}{n_{1}!\cdots n_{r}!} \end{array}$$

and if at least one of *n*_*i*_, *i* = 1,2,…, *r*, is odd; then

(51) $$\begin{array}{c} C_{n_{1},\ldots .,n_{r}}^{\left(\alpha \right)}\left(0,0,\ldots ,0\right)=0. \end{array}$$

**Proof**

If we set all *x*_*i*_ = 0, *i* = 1,2,…, *r* in (44), we have

(52) $$\begin{array}{c} {\left(1+t_{1}^{2}+\cdots +t_{r}^{2}\right)}^{-\alpha }=\sum_{n_{1},\ldots ,n_{r}=0}^{\infty }C_{n_{1},\ldots ,n_{r}}^{\left(\alpha \right)}\left(0,0,\ldots ,0\right)t_{1}^{n_{1}}\cdots t_{r}^{n_{r}}. \end{array}$$

On the other hand, we get

(53) (1+t12+⋯+tr2)−α =∑n1,…,nr=0∞(−1)n1+⋯+nrn1!⋯nr!(α)n1+⋯+nrt12n1⋯tr2nr.

By comparing the coefficients of *t*_1_^*n*_1_^ ⋯ *t*_*r*_^*n*_*r*_^, we obtain the desired.

From the theorems and corollaries given in Section 4, we can give some other properties of *C*_*n*_1_,…,*n*_*r*__^(*α*)^(*x*_1_,…, *x*_*r*_).

**Remark 14**

By Corollaries 8 and 9, for the family of multivariable polynomials generated by (44), the following relations:

(54) niCn1,…,nr(α)(x1,…,xr)  =xi∂∂xiCn1,…,nr(α)(x1,…,xr)   −∂∂xiCn1,…,ni−1,ni−1,ni+1,…,nr(α)(x1,…,xr),∑i=1rniCn1,…,nr(α)(x1,…,xr)  =∑i=1rxi∂∂xiCn1,…,nr(α)(x1,…,xr)   −∑i=1r∂∂xiCn1,…,ni−1,ni−1,ni+1,…,nr(α)(x1,…,xr)

hold for *n*_*i*_ ≥ 1,  *i* = 1,2,…, *r*.

**Remark 15**

From Theorem 11, the multivariable polynomials *C*_*n*_1_,…,*n*_*r*__^(*α*)^(*x*_1_,…, *x*_*r*_) satisfy the following addition formula:

(55) Cn1,…,nr(α+β)(x1,…,xr)=∑k1=0n1⋯∑kr=0nrCn1−k1,…,nr−kr(α)(x1,…,xr)       ×Ck1,…,kr(β)(x1,…,xr).

**Remark 16**

As a result of Theorem 12, expansions of *C*_*n*_1_,…,*n*_*r*__^(*α*)^(*x*_1_,…, *x*_*r*_) in series of Legendre, Gegenbauer, Hermite, and Laguerre polynomials are as follows:

(56) Cn1,…,nr(α)(x1,…,xr)  =∏i=1r{∑ki=0[ni/2]∑si=0[(ni−2ki)/2](2ni−4ki−4si+1)ki!si!(3/2)ni−2ki−si       ×(−1)kiPni−2ki−2si(xi)}     ×(α)n1+⋯+nr−k1−⋯−kr,Cn1,…,nr(α)(x1,…,xr)  =∏i=1r{∑ki=0[ni/2]∑si=0[(ni−2ki)/2](νi+ni−2ki−2si)ki!si!(νi)ni−2ki−si+1       ×(−1)kiCni−2ki−2siνi(xi)}     ×(α)n1+⋯+nr−k1−⋯−kr,Cn1,…,nr(α)(x1,…,xr)  =∏i=1r{∑ki=0[ni/2]∑si=0[(ni−2ki)/2](−1)kiHni−2ki−2si(xi)ki!si!(ni−2ki−2si)!}      ×(α)n1+⋯+nr−k1−⋯−kr,Cn1,…,nr(α)(x1,…,xr)  =∏i=1r{∑ki=0[ni/2] ∑si=0ni−2ki(βi+1)ni−2ki2ni−2ki(−1)siki!(ni−2ki−si)!(βi+1)si×(−1)kiLsi(βi)(xi)}    ×(α)n1+⋯+nr−k1−⋯−kr.

We now give a hypergeometric representation for the multivariable polynomials *C*_*n*_1_,…,*n*_*r*__^(*α*)^(*x*_1_,…, *x*_*r*_) given by (44).

**Theorem 17**

The multivariable polynomials *C*_*n*_1_,…,*n*_*r*__^(*α*)^(*x*_1_,…, *x*_*r*_) have the following hypergeometric representation:

(57) Cn1,…,nr(α)(x1,…,xr)  =(α)n1+⋯+nrn1!⋯nr!(2x1)n1⋯(2xr)nr   ×FB(r)[−n12,…,−nr2,−n1+12,…,−nr+12;       1−α−n1−⋯−nr;1x12,…,1xr2],         (max⁡{1x12,…,1xr2}<1),

where *F*_*B*_^(*r*)^ is a Lauricella function of *r*-variable defined by [13] (see also [2])

(58) FB(r)[a1,…,ar,b1,…,br;c;x1,…,xr]  =∑n1,…,nr=0∞(a1)n1⋯(ar)nr(b1)n1⋯(br)nr(c)n1+⋯+nrx1n1n1!⋯xrnrnr!,(max⁡{|x1|,…,|xr|}<1).

**Proof**

We have the following results from [11]:

(59) $$\begin{array}{c} {\left(\alpha \right)}_{n-k}=\frac{{\left(-1\right)}^{k}{\left(\alpha \right)}_{n}}{{\left(1-\alpha -n\right)}_{k}},\;\;{\left(-n\right)}_{2k}=2^{2k}{\left(-\frac{n}{2}\right)}_{k}{\left(\frac{-n+1}{2}\right)}_{k},\\ \left(n-2k\right)!=\frac{n!}{{\left(-n\right)}_{2k}}. \end{array}$$

In view of these relations, the equality (45) can be written as follows:

(60) Cn1,…,nr(α)(x1,…,xr) =∑k1=0[n1/2]⋯∑kr=0[nr/2](((α)n1+⋯+nr22(k1+⋯+kr)(−n12)k1‍×(−n1+12)k1⋯(−nr2)kr×(−nr+12)kr)×(k1!⋯kr!n1!⋯nr!×(1−α−n1−⋯−nr)k1+⋯+kr)−1)        ×(2x1)n1−2k1⋯(2xr)nr−2kr =(α)n1+⋯+nrn1!⋯nr!(2x1)n1⋯(2xr)nr  ×∑k1=0[n1/2]⋯∑kr=0[nr/2](((−n12)k1(−n1+12)k1             ⋯(−nr2)kr(−nr+12)kr)             ×((1−α−n1−⋯−nr)k1+⋯+kr               ×k1!⋯kr!)−1)          ×(1x12)k1⋯(1xr2)kr =FB(r)[−n12,…,−nr2,−n1+12,…,−nr+12;     1−α−n1−⋯−nr;1x12,…,1xr2]  ×(α)n1+⋯+nrn1!⋯nr!(2x1)n1⋯(2xr)nr,

where max⁡{1/*x*_1_^2^,…, 1/*x*_*r*_^2^} < 1. The proof is completed.

**Remark 18**

For *r* = 2, Theorem 17 reduces to the known result for two variable Gegenbauer polynomials given by [12].

### 5.2. The Case of *x*_*i*_ = 0, *y*_*i*_ = −*x*_*i*_, *i* = 1,2,…, *r* in (2)

This case yields

(61) (1−x1t1m1−x2t2m2−⋯−xrtrmr)−α  =∑n1,…,nr=0∞Un1,…,nr(α)(x1,…,xr)t1n1⋯trnr,

which is a different unification from that in [8].

**Remark 19**

From Corollaries 8 and 9, the multivariable polynomials *U*_*n*_1_,…,*n*_*r*__^(*α*)^(*x*_1_,…, *x*_*r*_) satisfy

(62) mixi∂∂xiUn1,…,ni−1,ni−mi+1,ni+1,…,nr(α)(x1,…,xr) =(ni−mi+1)Un1,…,ni−1,ni−mi+1,ni+1,…,nr(α)(x1,…,xr),∑i=1rmixi∂∂xi  ×Un1,…,ni−1,ni−mi+1,ni+1,…,nr(α)(x1,…,xr) =∑i=1r(ni−mi+1)    ×Un1,…,ni−1,ni−mi+1,ni+1,…,nr(α)(x1,…,xr).

for *n*_*i*_ ≥ *m*_*i*_ − 1, *i* = 1,2,…, *r*; *n*_*j*_ ≥ 0, *j* ≠ *i*.

**Remark 20**

As result of Theorem 11, the multivariable polynomials *U*_*n*_1_,…,*n*_*r*__^(*α*)^(*x*_1_,…, *x*_*r*_) have the following addition formula:

(63) Un1,…,nr(α+β)(x1,…,xr)=∑k1=0n1⋯∑kr=0nrUn1−k1,…,nr−kr(α)(x1,…,xr)       ×Uk1,…,kr(β)(x1,…,xr).

#### 5.2.1. The Case of *m*_*i*_ = 1, *i* = 1,2,…, *r* in Section 5.2

In this case, we get a *r*-variable analogue of Lagrange polynomials (see [14]) which is different from that in [10]

(64) (1−x1t1−⋯−xrtr)−α  =∑n1,…,nr=0∞Gn1,…,nr(α)(x1,…,xr)t1n1⋯trnr.

They are given explicitly by

(65) Gn1,…,nr(α)(x1,…,xr)=(α)n1+⋯+nrn1!⋯nr!x1n1⋯xrnr.

**Remark 21**

From Corollaries 8 and 9, the multivariable polynomials *G*_*n*_1_,…,*n*_*r*__^(*α*)^(*x*_1_,…, *x*_*r*_) satisfy

(66) $$\begin{array}{c} n_{i}G_{n_{1},\ldots ,n_{r}}^{\left(\alpha \right)}\left(x_{1},\ldots ,x_{r}\right)=x_{i}\frac{\partial }{\partial x_{i}}G_{n_{1},\ldots ,n_{r}}^{\left(\alpha \right)}\left(x_{1},\ldots ,x_{r}\right) \end{array}$$

for *i* = 1,2,…, *r* and

(67) ∑i=1rniGn1,…,nr(α)(x1,…,xr)  =∑i=1rxi∂∂xiGn1,…,nr(α)(x1,…,xr).

**Remark 22**

By Theorem 11, the multivariable polynomials *G*_*n*_1_,…,*n*_*r*__^(*α*)^(*x*_1_,…, *x*_*r*_) have the following addition formula:

(68) Gn1,…,nr(α+β)(x1,…,xr)=∑k1=0n1⋯∑kr=0nrGn1−k1,…,nr−kr(α)(x1,…,xr)×Gk1,…,kr(β)(x1,…,xr).

**Remark 23**

From Theorem 12, expansions of *G*_*n*_1_,…,*n*_*r*__^(*α*)^(*x*_1_,…, *x*_*r*_) in series of Legendre, Gegenbauer, Hermite, and Laguerre polynomials are given by

(69) Gn1,…,nr(α)(x1,…,xr)  =∏i=1r{∑si=0[ni/2](2ni−4si+1)si!(3/2)ni−siPni−2si(xi2)}     ×(α)n1+⋯+nr,Gn1,…,nr(α)(x1,…,xr)  =∏i=1r{∑si=0[ni/2](νi+ni−2si)si!(νi)ni−si+1Cni−2siνi(xi2)}     ×(α)n1+⋯+nr,Gn1,…,nr(α)(x1,…,xr)  =∏i=1r{∑si=0[ni/2]Hni−2si(xi/2)si!(ni−2si)!}(α)n1+⋯+nr,Gn1,…,nr(α)(x1,…,xr) =∏i=1r{∑si=0ni(βi+1)ni2ni(−1)si(ni−si)!(βi+1)siLsi(βi)(xi2)}    ×(α)n1+⋯+nr.

We can discuss some generating functions for the multivariable polynomials *G*_*n*_1_,…,*n*_*r*__^(*α*)^(*x*_1_,…, *x*_*r*_).

**Theorem 24**

For the polynomials *G*_*n*_1_,…,*n*_*r*__^(*α*)^(*x*_1_,…, *x*_*r*_),   the following generating function holds true for *k* ∈ *ℕ*_0_:

(70) ∑n1,…,nr=0∞Gn1,…,nr(α)(x1,…,xr)[k+ni]mz1n1⋯zrnr  =m!(1−x1z1−⋯−xrzr)−α    ×
g
 m(α,−k)(xizi1−x1z1−⋯−xrzr,−1),

for each *i* = 1,2,…, *r*, where

(71) $$\begin{array}{c} {\left[k\right]}_{m}:=k\left(k-1\right)\cdots \left(k-m+1\right),\;m\in \mathbb{N},{\left[k\right]}_{0}=1 \end{array}$$

and g _*n*_^(*α*,*β*)^(*x*, *y*) denotes the classical Lagrange polynomials defined by [14]

(72) $$\begin{array}{c} {\left(1-xt\right)}^{-\alpha }{\left(1-yt\right)}^{-\beta }=\sum_{n=0}^{\infty }{\;\text{ g }\;}_{n}^{\left(\alpha ,\beta \right)}\left(x,y\right)t^{n}\\ \left(\alpha ,\beta \in \mathbb{C};\left|t\right|<\min\left\{{\left|x\right|}^{-1},{\left|y\right|}^{-1}\right\}\right). \end{array}$$

**Proof**

Fix *i* = 1,2,…, *r*. Let *𝒞*_*ξ*_*i*__and *𝒞*_*ξ*_i__* denote the circles of radius *ɛ*_*i*_, centered *ξ*_*i*_ = *z*_*i*_ and *ξ*_*i*_ = 0, respectively, where

(73) $$\begin{array}{c} 0<{ɛ}_{i}<{\left|x_{i}\right|}^{-1}\left|1-\sum_{k=1}^{r}x_{k}z_{k}\right|. \end{array}$$

Here these circles are described in the positive direction. By Cauchy's Integral Formula, we have

(74) Dzim{(1−x1z1−⋯−xrzr)−αzik} =m!2πi∮𝒞ξiξik(1−x1z1−⋯−xiξi−⋯−xrzr)−α(ξi−zi)m+1dξi =m!2πi  ×∮𝒞ξi∗(ξi+zi)k(1−x1z1−⋯−xi(ξi+zi)−⋯−xrzr)−αξim+1dξi =m!2πizik(1−x1z1−⋯−xrzr)−α  ×∮𝒞ξi∗(1+ξi/zi)kξim+1(1−xiξi1−x1z1−⋯−xrzr)−αdξi =m!zik(1−x1z1−⋯−xrzr)−α  ×gm(α,−k)(xi1−x1z1−⋯−xrzr,−1zi) =m!zik−m(1−x1z1−⋯−xrzr)−α  ×gm(α,−k)(xizi1−x1z1−⋯−xrzr,−1).

On the other hand, we can write that

(75) Dzim{(1−x1z1−⋯−xrzr)−αzik} =Dzim{zik∑n1,…,nr=0∞Gn1,…,nr(α)(x1,…,xr)z1n1⋯zrnr} =∑n1,…,nr=0∞Gn1,…,nr(α)(x1,…,xr)dmdzimz1n1⋯zini+k⋯zrnr =zik−m∑n1,…,nr=0∞Gn1,…,nr(α)(x1,…,xr)[k+ni]mz1n1⋯zrnr.

From (74) and (75), we see that

(76) ∑n1,…,nr=0∞Gn1,…,nr(α)(x1,…,xr)[k+ni]mz1n1⋯zrnr =m!(1−x1z1−⋯−xrzr)−α  ×gm(α,−k)(xizi1−x1z1−⋯−xrzr,−1).

**Corollary 25**

From (71), one observes that

(77) [ni+m]m=(ni+m)(ni+m−1)⋯(ni+1)=(ni+1)m

for each *i* = 1,2,…, *r*. By setting *k* = *m*  in (70) and then using (77), one has

(78) ∑n1,…,nr=0∞(ni+1)mGn1,…,nr(α)(x1,…,xr)z1n1⋯zrnr =m!(1−x1z1−⋯−xrzr)−α  ×
g
 m(α,−m)(xizi1−x1z1−⋯−xrzr,−1).

#### 5.2.2. The Case of *m*_*i*_  =  *i*,**** *i*  =  1,2,…, *r* in Section 5.2

This case reduces to a multivariable analogue of Lagrange-Hermite polynomials

(79) (1−x1t1−x2t22−⋯−xrtrr)−α  =∑n1,…,nr=0∞Hn1,…,nr(α)(x1,…,xr)t1n1⋯trnr,

which is different from that in [6].

**Remark 26**

By Corollaries 8 and 9, the multivariable polynomials *H*_*n*_1_,…,*n*_*r*__^(*α*)^(*x*_1_,…, *x*_*r*_) satisfy

(80) ixi∂∂xiHn1,…,ni−1,ni−i+1,ni+1,…,nr(α)(x1,…,xr)  =(ni−i+1)Hn1,…,ni−1,ni−i+1,ni+1,…,nr(α)(x1,…,xr),∑i=1rixi∂∂xiHn1,…,ni−1,ni−i+1,ni+1,…,nr(α)(x1,…,xr)  =∑i=1r(ni−i+1)     ×Hn1,…,ni−1,ni−i+1,ni+1,…,nr(α)(x1,…,xr).

for *n*_*i*_ ≥ *i* − 1,  *i* = 1,2,…, *r*; *n*_*j*_ ≥ 0, *j* ≠ *i*.

**Remark 27**

From Theorem 11, the multivariable polynomials *H*_*n*_1_,…,*n*_*r*__^(*α*)^(*x*_1_,…, *x*_*r*_) have the following addition formula:

(81) Hn1,…,nr(α+β)(x1,…,xr) =∑k1=0n1⋯∑kr=0nrHn1−k1,…,nr−kr(α)(x1,…,xr)        ×Hk1,…,kr(β)(x1,…,xr).

Furthermore, setting *m*_*i*_ = *i*, *x*_*i*_ = 0, *y*_*i*_ = −*x*_*i*_, *i* = 1,2,…, *r* in Corollary 5, we obtain the following class of bilinear generating functions for the polynomials *H*_*n*_1_,…,*n*_*r*__^(*α*)^(*x*_1_,…, *x*_*r*_).

**Remark 28**

If

(82) Ξλ,η,α,β,γn,p(x1,…,xr;u1,…,ur;w) :=∑k1=0[n1/p1]⋯∑kr=0[nr/pr]ak1,…,krHn1−p1k1,…,nr−prkr(α+β)(x1,…,xr)×Hλ1+η1k1,…,λr+ηrkr(γ)(u1,…,ur)w1k1⋯wrkr,

where *a*_*k*_1_,…,*k*_*r*__ ≠ 0; *p*_*i*_ ∈ *ℕ*; *n*_*i*_, *λ*_*i*_, *η*_*i*_ ∈ *ℕ*_0_, *i* = 1,2,…, *r*, and *λ* = (*λ*_1_,…, *λ*_*r*_), *η* = (*η*_1_,…, *η*_*r*_),**w** = (*w*_1_,…, *w*_*r*_), then we have

(83) ∑l1=0n1⋯∑lr=0nr ∑k1=0[l1/p1]⋯∑kr=0[lr/pr]ak1,…,krHn1−l1,…,nr−lr(α)(x1,…,xr)            ×Hl1−p1k1,…,lr−prkr(β)(x1,…,xr)            ×Hλ1+η1k1,…,λr+ηrkr(γ)(u1,…,ur)            ×w1k1⋯wrkr =Ξλ,η,α,β,γn,p(x1,…,xr;u1,…,ur;w).
